# *Pseudomonas otitidis* bacteremia in an immunocompromised patient with cellulitis: case report and literature review

**DOI:** 10.1186/s12879-023-08919-0

**Published:** 2023-12-18

**Authors:** Takeo Mori, Sadako Yoshizawa, Kageto Yamada, Takahiro Sato, Masakazu Sasaki, Yusuke Nakamura, Ukyo Gen, Hinako Murakami, Katsuhito Kashiwagi, Tadashi Maeda, Taito Miyazaki, Tetsuo Yamaguchi, Yoshihisa Urita, Yoshikazu Ishii, Kazuhiro Tateda

**Affiliations:** 1 Department of General Medicine and Emergency Care (Infectious Diseases), Toho University Omori Medical Center, 6-11-1 Omorinishi, Ota-ku, Tokyo, 143-8541 Japan; 2 Department of Microbiology and Infectious Diseases, Toho University School of Medicine, 5-21-16 Omorinishi, Ota-ku, Tokyo, 143-8540 Japan; 3 Department of Clinical Laboratory, Toho University Omori Medical Center, 6-11-1 Omorinishi, Ota-ku, Tokyo, 143-8541 Japan; 4 Department of Laboratory Medicine, Toho University School of Medicine, 5-21-16 Omorinishi, Ota-ku, Tokyo, 143-8540 Japan; 5 Department of Orthopaedic Surgery, Toho University Omori Medical Center, 6-11-1 Omorinishi, Ota-ku, Tokyo, 143-8541 Japan; 6 Department of General Medicine and Emergency Care, Toho University School of Medicine, 6-11-1 Omorinishi, Ota-ku, Tokyo, 143-8541 Japan

**Keywords:** *Pseudomonas otitidis*, Bacteremia, Cellulitis, Case report, Immunocompromised patient, POM-1

## Abstract

**Background:**

*Pseudomonas otitidis* belongs to *the genus Pseudomonas* and causes various infections, including ear, skin, and soft tissue infections. *P. otitidis* has a unique susceptibility profile, being susceptible to penicillins and cephalosporins but resistant to carbapenems, due to the production of the metallo-β-lactamase called POM-1. This revealed genetic similarities with *Pseudomonas aeruginosa*, which can sometimes lead to misidentification.

**Case presentation:**

We report the case of a 70-year-old Japanese male who developed cellulitis and bacteremia during chemotherapy for multiple myeloma. He was initially treated with meropenem, but blood culture later revealed gram-negative bacilli identified as *P. otitidis* using matrix-assisted laser desorption ionization-time of flight mass spectrometry (MALDI-TOF MS). Carbapenem resistance was predicted from previous reports; therefore, we switched to dual therapy with levofloxacin and cefepime, and favorable treatment results were obtained.

**Conclusion:**

This is the first reported case of *P. otitidis* cellulitis and bacteremia in an immunocompromised patient. Carbapenems are typically used in immunocompromised patients and *P. otitidis* is often resistant to it. However, its biochemical properties are similar to those of *Pseudomonas aeruginosa*; therefore, its accurate identification is critical. In the present study, we rapidly identified *P. otitidis* using MALDI-TOF MS and switched from carbapenems to an appropriate antimicrobial therapy, resulting in a successful outcome.

## Background

*Pseudomonas otitidis* is a species of *Pseudomonas* bacteria that was first registered as a new species in the United States in 2006 and was initially reported as a cause of ear infections [1]. In recent years, it has been reported to cause more severe infections [2, 3], and in 2021, bacteremia caused by *P. otitidis* was reported in an immunocompetent patient with COPD [4]. *P. otitidis* has characteristic drug susceptibility, showing sensitivity to penicillins and cephalosporins, while exhibiting a tendency for resistance to carbapenems due to the production of a species-specific metallo-β-lactamase, named POM-1 (*P. otitidis* metallo-β-lactamase) [5]. Early diagnosis of *P. otitidis* is extremely important in immunocompromised patients with febrile neutropenia, because of carbapenems are often used as initial treatment. Here, we describe a patient who developed cellulitis and bacteremia caused by *P. otitidis* during chemotherapy for multiple myeloma. To the best of our knowledge, this is the first reported case of bacteremia in an immunocompromised patient caused by *P. otitidis*.

## Case presentation

A 70-year-old Japanese male who is currently receiving outpatient chemotherapy for multiple myeloma at another hospital was admitted to our hospital for lower leg edema and nephrotic syndrome. His medical history included multiple myeloma (Durie-Salmon stage III), chronic kidney disease, type 2 diabetes mellitus (30 years), hypertension, dyslipidemia, benign prostatic hyperplasia, psoriasis vulgaris, and herpes zoster infection. Chemotherapy was administered every 5 weeks for multiple myeloma, and the most recent course was one month prior (course 26). The chemotherapy regimen consisted of oral administration of dexamethasone 10 mg on days 1, 2, 8, and 9; pomalidomide from day to 1–14, and bortezomib on days 1 and 8. He was administered with sulfamethoxazole 400 mg/trimethoprim 80 mg once daily to prevent *Pneumocystis* pneumonia. He had no known allergies to medication or food.

At the time of admission, there were no subjective symptoms; however, on the fourth day of hospitalization, the patient presented with fever, chills, and pain at the right thigh. The physical examination findings are as follows: body temperature of 36.9 °C, heart rate of 65 beats per minute, respiratory rate of 18 breaths per minute, and blood pressure of 120/66 mmHg. The patient was in good general condition. Faint erythema, swelling, warmth, and tenderness were observed in the right thigh and front of the lower leg. Crepitations were not observed. No petechiae, purpura, bullae, or vesicles were observed. Edema with indentation was observed in both lower legs. No other physical abnormalities, including findings suggestive of endocarditis, were observed.

The findings of the blood and urine examinations are summarized in Table 1. Computerized tomography scans did not reveal any abnormalities in the lungs, liver, gallbladder, pancreas, spleen, or intestine. The lower extremities were not included in the CT scan.

**Table 1:** Results of blood and urine tests on the fourth day of hospitalization with fever

Blood test			Urine test (qualitative)	
White blood cell	1.3	× 10^3^/μl	Glucose	3+
Red blood cell	3.57	× 10^6^/μl	Protein	3+
Haemoglobin	10	g/dl	Occult blood	2+
Hematocrit	29.6	%	Urinary sediments	
Platelet	109	×10^3^/μl	White blood cell (Cell/ High Power Field)	10–19
Basophil	0	%	Red blood cell (Cell/High Power Field)	1–4
Eosinophil	0	%		
Lymphocyte	18.7	%		
Monocyte	18.7	%		
Neutrophil	62.6	%		
C-reactive protein	5.6	mg/L		
Serum sodium	141	mEq/L		
Serum potassium	3.2	mEq/L		
Serum chloride	107	mEq/L		
Urea nitrogen	23	mg/dL		
Creatinine	1.61	mg/dL		
Urine acid	5.5	mg/dL		
Aspartate aminotransferase	31	U/l		
Alanine aminotransferase	20	U/l		
Lactate Dehydrogenase	333	U/l		
Alkaline Phosphatase	48	U/l		
Glutamyl transpeptidase	15	U/l		
Creatine kinase	157	U/dl		
Hemoglobin A1c	5.9	%		
Prothrombin time	147	%		
Activated partial thromboplastin time	27.3	sec		
Fibrinogen quantity	741	mg/dL		
D-dimer	6.1	μg/mL		
Immunoglobulin G	313	mg/dL		
Immunoglobulin A	57	mg/dL		
Immunoglobulin M	11	mg/dL		

It was considered that there are humoral and cellular immunodeficiencies due to multiple myeloma and dexamethasone administration. Although it is difficult to calculate an accurate LRINEC score because blood glucose levels were not measured, the highest estimate was 5 points, then cellulitis was more likely based on clinical findings [6]. Owing to the low neutrophil count and the prospective further decline, meropenem was initiated for febrile neutropenia after blood cultures were submitted.

**Fig. 1:**
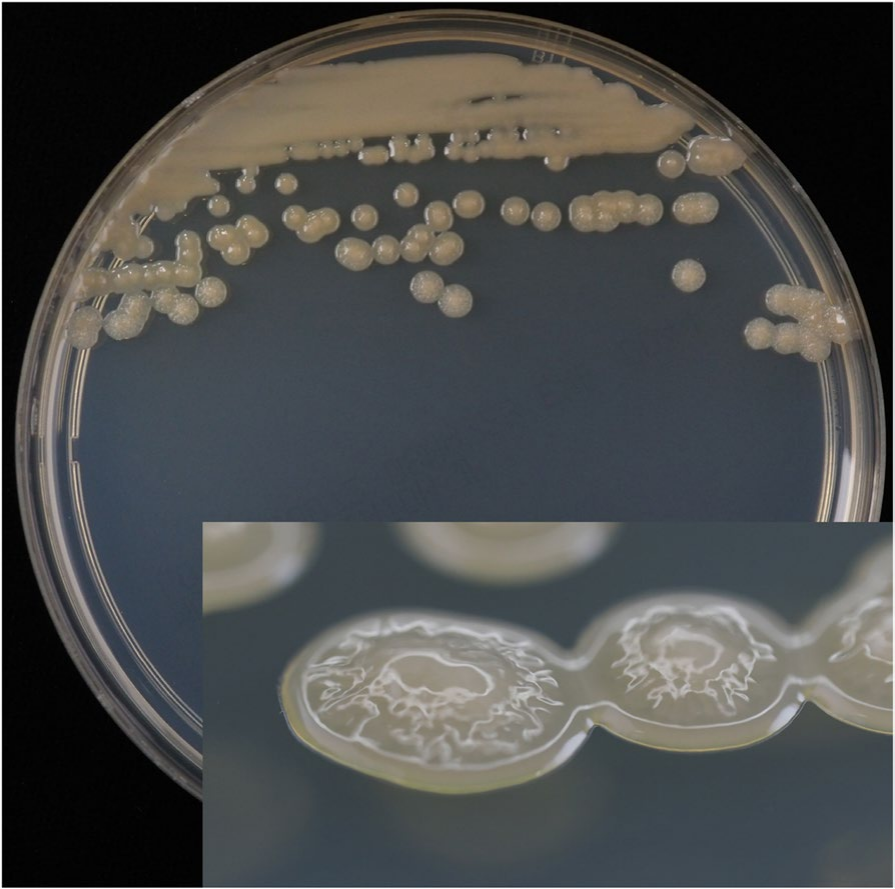
*Pseudomonas otitidis* colony morphology isolated in this case, 24-h colony on Mueller Hinton agar

The next day, gram-negative rods were detected in two sets of aerobic blood culture bottles. Bacterial colonies were initially small, smooth, and wrinkled after 24 h of incubation (Fig. 1). Biochemical characteristics were determined using the DxM Microscan WalkAway system (Beckman Coulter, Brea, CA, USA), which identified *P. putida/fluorescens* based on a high identification score of 99.99%. Conversely, identification of the colony using Matrix-assisted laser desorption/ionization time-of-flight mass spectrometry (MALDI-TOF MS; Bruker Daltonics GmbH, Bremen, Germany) revealed *P. otitidis* with a score of 2.29.

Carbapenem resistance was predicted based on previous reports on *P. otitidis*, and meropenem was substituted with cefepime and levofloxacin. Antibiotic susceptibilities were determined according to the Clinical and Laboratory Standards Institute(CLSI) M07 tenth edition and M100 26th edition using the automated system of the DxM MicroScan WalkAway System (Beckman Coulter, USA) [7, 8]. The results of the antimicrobial susceptibility testing are shown in Table 2. Although MIC of meropenem was found to be high and that of imipenem was relatively high (4 μg/mL), the MIC of penicillin and cephalosporin antibiotics were found to be low. The ciprofloxacin MIC was low (Table 2). The production of carbapenemase and MBL was confirmed using the modified carbapenem inactivation method and the double disk synergy test with sodium mercaptoacetate, respectively [8, 9]. Combination therapy with cefepime and levofloxacin was administered for 2 weeks to avoid the acquisition of resistance to levofloxacin during the two-week treatment period for bacteremia. Blood cultures obtained on the 5th day after the first positive blood culture were negative. The patient’s condition improved and there was no recurrence of cellulitis or bacteremia thereafter.

**Table 2:** Results of drug susceptibility testing for isolated *P. otitidis*. It showed resistance to meropenem, while being susceptible to penicillin, cephalosporin, aminoglycoside, and quinolone

Antibiotics	MIC (μg/mL)
Piperacillin	<= 8
Sulbactam/Ampicillin	> 32
Tazobactam/piperacillin	<= 8
Ceftazidime	<= 4
Cefepime	<= 4
Imipenem	4
Meropenem	> 8
Gentamicin	<= 1
Amikacin	<= 4
Minocycline	2
Ciprofloxacin	<= 0.25
Aztreonam	4
Sulfamethoxazole-Trimethoprim	<= 20

## Discussion

*P. otitidis* is a bacterium belonging to the *Pseudomonas* genus and was first identified as a pathogen associated with human ear infections in the United States in 2006 [1]. Initially reported as a causative agent of ear infections, such as acute and chronic otitis media, and acute otitis externa, recent reports have also shown associations with more invasive diseases, such as epididymo-orchitis, necrotizing fasciitis, and diffuse peritonitis [1–3], although clinical reports are limited. In 2021, the first case of bacteremia was reported [4]. Previous reports indicate its environmental distribution in Nigeria and Lake Kawaguchi in Japan [10, 11], and concerns have been raised regarding its potential to cause infections in immunocompromised patients and more severe presentations [2]. To the best of our knowledge, this is the first case report of bacteremia caused by *P. otitidis* in an immunocompromised patient, highlighting the potential risks of severe infections in such patients.

*P. otitidis* produces chromosomally encoded POM-1, which efficiently hydrolyzes carbapenem and penicillin antibiotics, but weakly hydrolyzes cephalosporin antibiotics [12]. POM-1 is similar to the PAM-like MBL produced by *P. tohonis* and L1-like MBL produced by *S. maltophilia,* with homologies of 72–73% and 60–64% in amino acid sequences, respectively [5, 13]. In addition to the production of POM-1, *P. otitidis* may acquire resistance to carbapenems through inoculum size effect, decreased outer membrane permeability, and upregulation of the efflux system, whereas it does not possess AmpC β-lactamase [5]. These factors suggest that carbapenems should not be used when this organism is suspected.

In the present case, as in previous reports, the MICs of carbapenems were higher than those of other β-lactams and the MIC of ciprofloxacin was low. To avoid the acquisition of resistance, combination therapy with cefepime and levofloxacin was administered. In terms of antimicrobial agent choice, it is considered that POM-1 has high hydrolyzing efficiency for carbapenems and penicillin antibiotics but weak hydrolyzing efficiency for cephalosporin antibiotics [5]; cefepime was selected. The patient’s condition improved with the combination therapy. There is a report that *P. otitidis* does not produce AmpC β-lactamase, but according to the drug susceptibility results of 20 strains of *P. otitidis* in the same literature, the MIC value of ceftazidime was relatively high, we considered the influence of other mechanisms such as efflux pump, and decided on combination therapy. Although there is a lack of evidence supporting the need for combination therapy, further evidence is warranted for the treatment of this bacterial infection.

In terms of species identification, high genetic homology with *P. aeruginosa* has been observed, and the similarity of 16S rRNA genes has been reported to be 98.6% [1]. Identification based on colonies or phenotypes has been reported to be difficult [14]. In fact, the isolate from our case was identified as *P. putida/fluorescens* based on its biochemical characteristics using the DxM Microscan WalkAway system (Beckman Coulter, CA, USA), with a high identification score of 99.99%. According to a previous report, identification based on biochemical phenotypic characteristics can lead to misidentification of other *Pseudomonas* species [15]. It has been reported that *P. otitidis* may be misidentified as *P. putida/fluoressens* using the DxM Microscan WalkAway system. On the other hand, MALDI Biotyper has a higher identification rate equivalent to that of whole genome sequencing [15]. The possibility of misidentification has been pointed out, which could lead to the incorrect selection of antibacterial therapy. Therefore, it is necessary to consider the possibility of *P. otitidis* when the pathogen is suspected to be a *Pseudomonas* species and exhibits distinctive susceptibility, such as susceptibility to penicillin or cephalosporin antibiotics but resistance to carbapenems. Immunocompromised patients are often treated with carbapenems; therefore, more caution is needed regarding the possibility of *P. otitidis.*

## Conclusion

*P. otitidis* is often misidentified as other bacterial species by conventional identification methods. Due to production of POM-1 and resistance to carbapenems, caution is required in immunocompromised hosts, especially in bacteremia with febrile neutropenia. On the other hand, rapid diagnosis by MALDI-TOF MS has become possible in recent years. Therefore, when *P. otitidis* is suspected by MALDI-TOF MS, it is recommended to use antibiotics other than carbapenems.

## Data Availability

All data supporting the conclusions of this case report are included in the article. If additional data sets are required, please contact to t.mori@med.toho-u.ac.jp for further information.

## Abbreviations

MIC: Minimum inhibitory concentration
CT: Computed tomography
MALDI-TOF MS: Matrix-assisted laser desorption / ionization time-of-flight mass spectrometry
SMA: sodium mercaptoacetate
POM-1: *P. otitidis* metallo-β-lactamase-1
MBL: metallo-β-lactamase
